# Effect of milk feed source, frequency of feeding and age at turnout on calf performance, live-weight at mating and 1^st^ lactation milk production

**DOI:** 10.1186/2046-0481-65-18

**Published:** 2012-10-18

**Authors:** David Gleeson, Bernadette O’Brien

**Affiliations:** 1 Livestock Systems Department, Animal & Grassland Research and Innovation Centre, Teagasc, Moorepark, Fermoy, Co Cork, Ireland

**Keywords:** Calves, Feeding frequency, Calf performance, Outdoor rearing

## Abstract

Female calves (n = 108) were assigned to 6 cold milk feeding treatments in two experiments for a 70-day period. Live-weight (LW) was measured weekly, with an additional LW taken at day 410 and post-calving for animals in experiment 1. In Experiment 1, the effect of feeding frequency and age of turnout to pasture on calf performance and 1^st^ lactation milk yields were evaluated. The whole milk (WM) feeding treatments applied were (i) once daily feeding (OD), (ii) twice daily feeding (TD), (iii) OD feeding, outdoors at 38 days (ODO). In Experiment 2, the effects of feeding milk replacer (MR) as opposed to WM and age of turnout to pasture on calf performance were evaluated. The treatments applied were (i) OD feeding with WM (OD), (ii) OD feeding with milk replacer (MR) (ODMR), (iii) OD feeding with MR, outdoors at 38 days (ODMRO). Experiment 1: There were no differences (P > 0.05) in LW or average daily gain between TD and OD calves at day 80 or 410. ODO calves had lower LW at day 80 as compared to OD or TD (P < 0.001). Calf LW at day 80 was 86, 89 and 85 kg and at day 410 was 304, 309 and 316 kg for OD, TD and ODO, respectively. Milk feeding frequency or time of calf turnout had no effect on LW post calving, milk composition or 1st lactation milk yields. Experiment 2: Total LW at day 80 was higher (P < 0.05) for ODMR compared to OD or ODMRO calves. Calf LW was 87, 95, and 88 kg for OD, ODMR and ODMRO, respectively. However, LW at day 410 did not differ between treatments.

This study showed that while some differences were observed in calf LW at day 80, these differences had no effect on LW at day 410 or 1^st^ lactation milk yield. It can be concluded that calves can be successfully reared when fed OD with WM or MR, indoors and when turned out to pasture at 38 days of age.

## Background

Rearing the pre-weaned calf is one of the most challenging tasks on the dairy farm, particularly with a spring milk production system, where a high proporotion of calves are born over a 12-week period. With regard to female calves, the main objective is to rear a healthy calf to optimium mating weight [1] that will achieve optimium milk production in subsequent lactations. Since feed and labour are the two major inputs of calf rearing, it is important to establish the effect of alternative aspects of both on calf and cow performance. Calf feeding methods can influence labour input and calf performance [2]. The feeding of cold milk to calves once daily offers the opportunity for milk feeding to be carried out at an off-peak time during the working day. No difference in calf weight gain was observed when calves were individually fed cold milk replacer (room temperature) compared to warm milk (38°C) [3]. Feeding cold milk to calves has also been shown to increase concentrate intake and reduce milk intake [4]. Studies by Fallon et al. [5] have indicated that calves do not experience a severe nutrient deprivation on being introduced to once a day feeding regimne. In addition group feeding of calves once a day with cold whole milk indoors has been shown not to have any adverse affect on calf performance [6]. Furthermore, the labour requirement for calf care may be reduced if calves are grouped rather than reared in individual pens [2,7]. The grouping of calves has also been shown to have advantages in terms of weight gain after weaning over those fed individually [8]. Rearing calves outdoor appears to have benefits in terms of performance, labour and disease. Jorgenson et al. [9] found no difference in growth rates of calves reared outdoors in hutches compared to calves reared indoors in individual pens. They further suggested that a lower labour input was associated with the outdoor system. Calves can adapt to outdoor temperatures if a draft free area and dry bed is provided with adequate feed [10]. A lower incidence of respiratory cases could be expected for calves reared outdoors as compared to those reared indoors [11]. With an increase in the value of whole milk and concerns about the potential spread of Johne’s disease, there is also a renewed interest in the feeding of milk replacer. Randall and Swannack [12] demonstrated no differences between feeding of cold milk replacer compared to warm milk replacer. Calf performance was not affected by feeding milk replacer once daily in individual pens [13]. Most studies on once daily calf feeding have investigated the effect of feeding milk replacer to calves individually. There is limited knowledge on feeding milk replacer to calves as a group combined with early turnout on calf performance. The objective of the current study was to evaluate the effect of group feeding calves once daily with whole milk and milk replacer, indoors and outdoors, with respect to calf performance and live-weight at mating. The effect of the initial calf rearing system on 1^st^ lactation milk production was also investigated.

## Materials and methods

In Experiment 1, female calves (n = 54) were randomized and assigned to three whole milk (WM) feeding treatments, based on date of birth, calf live-weight (LW) and breed. Calf breed consisted of Holstein Friesan (78%) and Montebeliarde (22%). Calves were assigned to groups at an average age of 10 days (start day) and remained on treatment for 70 days. The average LW per calf at the start day (day 10) were 39.8 kg.The treatments applied were (i) once daily feeding (OD), (ii) twice daily feeding (TD) or (iii) OD feeding going outdoors at 38 days (ODO). At an average calf age of 38 days it was expected that calves would have an adequate live-weight to cope with the inclement spring weather conditions in late March. Treatment groups OD and TD remained indoors untill day 80 at which point they were turned out to grass.

In Experiment 2, female calves (n = 54) were assigned to one of three liquid feeding treatments using the same criteria as with Experiment 1. Calf breed consisted of Holstein Friesan (78%) and Montebeliarde (22%). The feeding treatments applied were (i) OD feeding with WM (OD), (ii) OD feeding with milk replacer (ODMR) or (iii) OD feeding with MR going outdoors at 38 days (ODMRO). Calves offered MR (Heiferlac, Volac, United Kingdom) received a mean daily allowance of 454 gms in 2.75 litres of water. The MR is promoted for the rearing of heifer calves to achieve fast frame growth in the early part of life and contains high levels of pure protein, lactose and vegetable oils (protein 26%, oil 16%, ash 7% and fibre 0%). Average calf LW at the start (day 10) was 39.3 kg. Treatment groups OD and ODMR remained indoors until day 80 and then located outdoors on grass.

**Figure 1:**
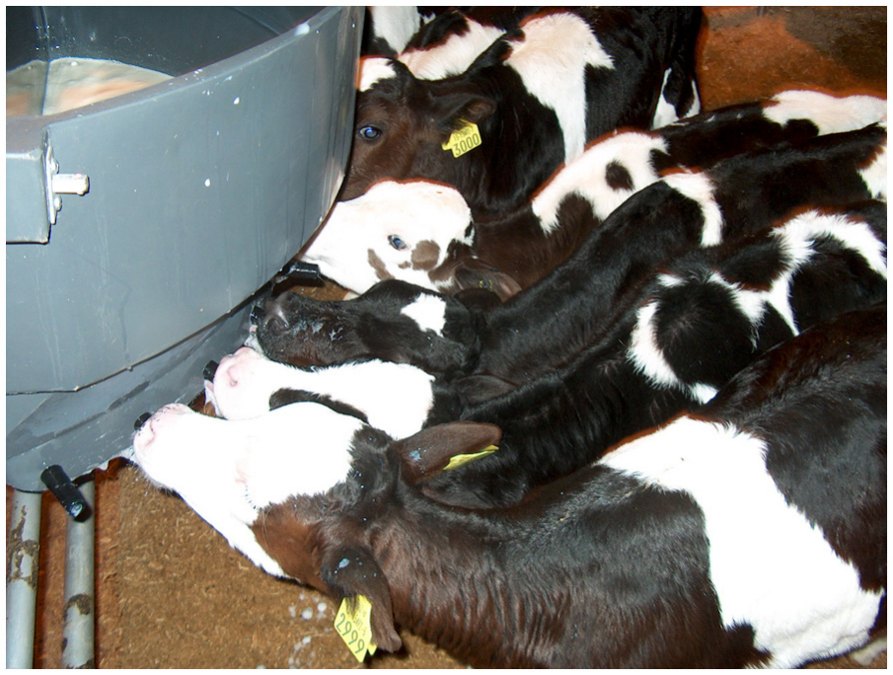
Calf milk feeding using a mobile teat feeder.

Experiments 1 and 2 were conducted in years 2007 and 2008, respectively, with a number of common calf rearing procedures used for both experiments. Calves on each treatment were penned in groups of nine and each treatment group was replicated. Group pens were filled before calves were assigned to the next treatment group. This method was used to assure equal age profile within pens and to maintain a stable number of calves within each pen. This method also facilitated weaning steps. All calves were fed twice daily with colostrum for 3 days and then whole milk until day 10. Calf feeding times were 0900 h for OD calves and calves fed TD were offered their second feed at 1530 h. Whole milk was collected daily, during milking for calf feeding, after plate cooling (milk temperature ranged from 10°C to 15°C). All calves were fed using mobile teat feeders (not individually sub-divided) suspended on the gate of each pen or paddock (Figure 1). After each feeding occasion milk teat feeders were removed and washed with running water. Calves, offered WM twice daily, received 2.25 liters of milk at each feeding time. Calves offered WM once daily received 4.5 liters of milk at the morning feeding time. Electrolytes were added to water and offered to calves where calf diarrhoea was diagnosed. Calves showing clinical signs of pneumonia and with a rectal temperature ≥ 40C were administered antibiotics.

**Figure 2:**
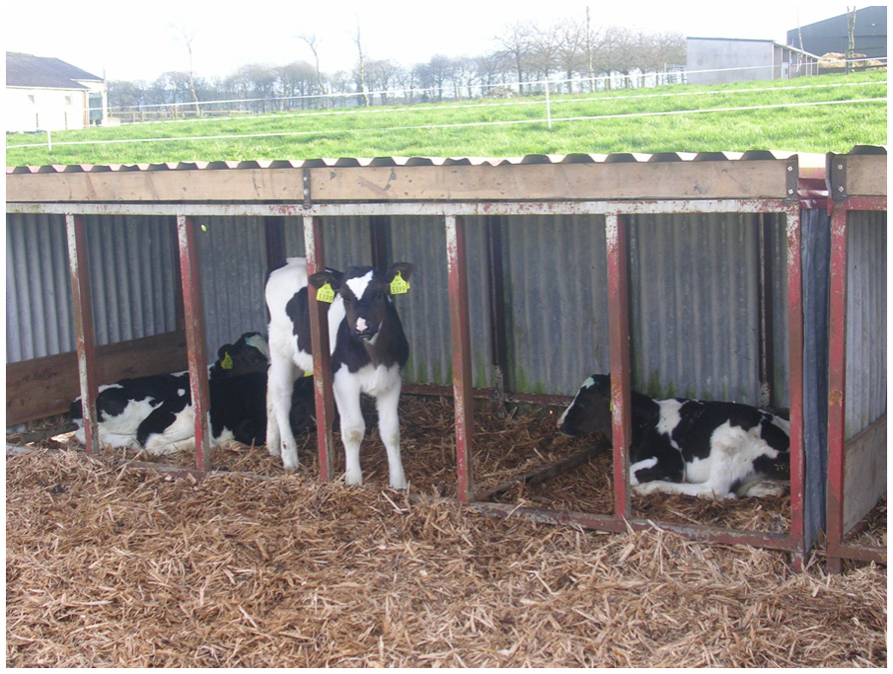
Mobile shelter for outdoor calf rearing.

Calves were weaned off milk at 66 days and had their total daily milk volume reduced gradually from 4.5 to 1 litre per day starting at day 60 until weaning. Calves were offered a calf starter ration ad libitum from day 10 until day 66 and a commercially available calf pellet ad libitum thereafter, until day 80. The calf starter ration contained barley, soya, flaked maize, sunflower seed, lucerne, calcium carbonate, beet pulp molasses, wheat, and sodium bicarbonate (protein 18%, oil 3.5%, fiber 6.0%, ash 8.0%, moisture 14%, vitamins A and D3). Calves were housed indoors in a naturally ventilated house. Calves were bedded using sawdust, with additional sawdust added if beds appeared soiled or wet. Self-filling fresh water troughs were located in each of the group pens or paddocks. The total indoor pen area available per calf was 2.62 m^2^. Perennial ryegrass hay was offered ad-lib to all calves indoors from day 38. A mobile calf shelter unit made of box iron with galavanized sheeting (360 cm x 215 cm ) was designed to offer shelter to calves outdoors from day 38 (Figure 2). The shelters which could be moved by mechanical loader were located in paddocks on a wood bark base and faced away from the prevailing wind.

### Measurements

Daily health parameters such as calf diarrhoea, group diarrhoea (entire pen showing clinical signs of diarrhoea), pneumonia and deaths were recorded up to day 80. Individual calf LW was recorded at the start day (day 10) and weekly thereafter until day 80 using a mobile weighing scales (Tru-test Ltd, Auckland, New Zealand). Animal LW results are presented forthnightly. In addition, maiden heifers were weighed on one occasion prior to start of the breeding season and individual heifer weights were calculated for day 410 based on the ADG from day 80 to the actual weigh day.

Weekly group intakes of concentrates were recorded with differences between pen groups measured during the period day 10 to 38. The range of difference in group concentrate intake between replicate pens was 0.09 and 0.14 kg for experiment 1 and 2, respectively. Group pens for each treatment were amalgamated at day 38 for calves indoors and outdoors. Treatment intakes of concentrates, hay and milk was recorded to day 80. The DM content were calculated for each feed type (whole milk, 12.4% solids, concentrate, 87% and Hay, 85%) to establish differences in the overall DM intake between feeding systems today 80. Grass allowance or DM intake were not measured.

Calves in Experiment 1 and 2 were managed on pasture with non-experimental animals from May to November (day 81 to day 261 approximately). Concentrates were fed (1.5 kg/h/d) after turnout for approximately six weeks. Calves in Experiment 1, were housed in November on cubicles and fed a maize silage diet with 1.5 kg/h/d of concentrates up to the start of the breeding season. Milk production and milk composition data was measured during the first lactation for those calves reared in Experiment 1, which had completed at least 200 lactation Days. The LW and body condition score (BCS) of those animals were also recorded post-calving (within one week of calving day). Animal BCS was measured on a scale of 1 to 5 (1 = extremely thin, 5 = fat) in increments of 0.25. Calves in Experiment 2, were managed on different indoor and outdoor forage feeding systems, during the following winter period [14].

### Statistical analysis

LW performance data were analyzed using mixed models with the Mixed procedure of SAS (SAS, 2006). Milk production data was analysed using mixed model procedures in SAS with repeated measures.

All experiments compiled with EU Council Directive 98/58/EC which concerns the protection of animals kept for farming purposes.

## Results

### Experiment 1

Differences in calf LW were observed at day 38 (P < 0.01), 52 (P < 0.001), 66 (P < 0.001) and 80 (P < 0.001) with ODO having lower LW than either OD or TD (Table 1). The total weight gain from day 10 to 80 was 47, 49 and 45 for OD, TD and ODO, respectively. Calf LW at day 80 was 86, 89 and 85 kg for OD, TD and ODO, respectively. The ODO calves had lower (P < 0.05) average daily gain (ADG) for period (day 25 to 38) and for period (day 39 to 52) (P < 0.01) compared to either OD or TD (Table 2). TD had higher ADG for the week (day 53 to 66) compared to OD or ODO. ODO had higher (P < 0.001) ADG during the week (day 67 to day 80) compared to either OD or TD. However, when ADG was calculated for days 10 to 80 and from day 81 to 410 there were no significant differences between feeding treatments.

**Table 1:** **Effect of milk feeding frequency and turnout age on calf live-weight from day 10 to 80 and atday 410-Experiment 1**

**Day**	**OD**	**TD**	**ODO**	**s**.**e**	**Sig**
10	39.8	40.4	39.4	2.2	NS
24	43.2	45.0	43.2	2.2	NS
38	53.5^a^	53.4^a^	49.4^b^	2.2	**
52	68.3^a^	66.8^a^	58.6^b^	2.2	***
66	76.3^a^	80.3^a^	66.8^b^	2.2	***
80	86.4^a^	89.1^a^	84.5^b^	2.2	***
410	304	309	316	5.2	NS

OD = once a day, TD = twice a day, ODO = once a day/outdoor.

**Table 2:** Effect of milk feeding frequency and turnout age on average daily weight gain (kg)-Experiment 1

**Day**	**OD**	**TD**	**ODO**	**s**.**e**	**Sig**
10-24	0.24	0.33	0.28	0.07	NS
25-38	0.74^a^	0.60^a^	0.44^b^	0.07	*
39-52	1.01^a^	0.96^a^	0.66^b^	0.07	**
53-66	0.57^a^	0.97^b^	0.59^a^	0.07	**
67-80	0.72^a^	0.63^a^	1.26^b^	0.08	***
10-80	0.67	0.69	0.64	0.03	NS
80-410	0.66	0.68	0.69	0.02	NS

OD = once a day, TD = twice a day, ODO = once a day/outdoor.

**Figure 3:**
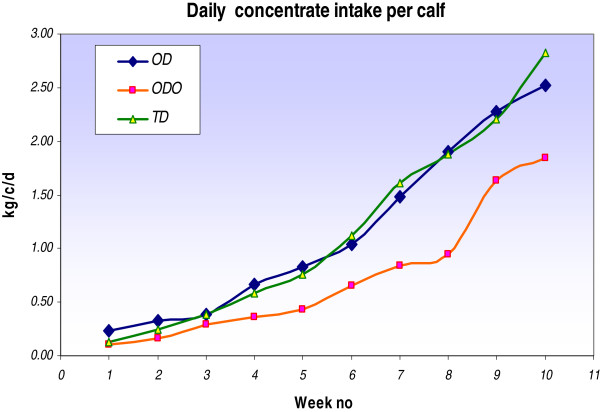
Calf intake of concentrates per day-Experiment 1.

The weekly concentrate intake and the total DM intake for milk, concentrate and hay for each feeding system is shown in Figure 3 and Table 3, respectively. The total DM intake per calf (10–80 days) was 108, 108, and 72 kg for OD, TD and ODO, respectively. Intakes of grass were not recorded for the ODO treatment. ODO had lower daily intakes of concentrates from turnout until day 80 (Figure 3). Four calves were culled from the OD group due to an unrelated health issue before day 80 and were not available for further measurements. Data for these animals were removed from the complete dataset. There were no differences in other health parameters measured between treatments for Experiment 1 (Table 4). In Experiment 1 the mean LW of heifers at 410 days (breeding start date) was 304, 309, and 316 kg and the ADG (day 80 to 410) was 0.66, 0.68 and 0.69 kg/d for OD, TD and ODO, respectively (Tables 1 and 2). There were no differences in body condition scores (BCS) or mean LW for treatment groups post-calving (Table 5). Cow LW was 464, 476, and 493 kg for OD, TD and ODO, respectively. The average 1^st^ lactation milk production and milk composition per cow did not differ between calf rearing treatments. Total milk production per cow was 4,450, 4,766, 4,789 kg for OD, TD and ODO, respectively.

**Table 3:** Dry Matter (DM) intake per calf (milk concentrate, hay) day 10 to day 80 - Experiment 1 and 2 (kg)

	**Experiment 1**			**Experiment 2**		
	**OD**	**TD**	**ODO**	**OD**	**ODMR**	**ODMRO**
Milk	28	28	28	28	22	22
Concentrate	71	71	44	61	89	65
Hay	8.5	8.8	0	5.5	5.9	0
DM per Kg LW gain	2.3	2.2	1.6^*^	2.0	2.1	1.6^*^

OD = once a day, TD = twice a day, ODO = once a day/outdoor.

ODMR = once a day/milk replacer, ODMRO = once a day/milk replacer/outdoor.

* contribution of grass not measured.

**Table 4:** Individual calf health events (day 10 to day80)- for Experiment 1 and 2

	**Experiment 1**			**Experiment 2**		
	**OD**	**TD**	**ODO**	**OD**	**ODMR**	**ODMRO**
Calf Diarrhoea	0	2	0	3	1	3
Pneumonia	0	2	1	8	1	6
Group Diarrhoea	1	0	0	0	0	0
Colic	0	0	0	0	0	1
Deaths	0	1	0	1	0	0

OD = once a day, TD = twice a day, ODO = once a day/outdoor.

ODMR = once a day/milk replacer, ODMRO = once a day/milk replacer/outdoor.

**Table 5:** **Effect of calf milk feeding frequency and turnout age on live-weight (kg) and body condition score (BCS) post calving and on 1**^**st **^**lactation milk production performance-Experiment 1**

**Treat**	**Milk yield(kg)**	**SCM**	**Fat(kg)**	**Fat%**	**Protein(kg)**	**Protein%**	**Lactose(kg)**	**Lactose%**	**B/wgt**	**BCS**
OD	4,450	352	195	4.39	157	3.5	210	4.72	464	4.1
TD	4,766	371	212	4.46	170	3.6	225	4.72	476	4.3
ODO	4,789	382	205	4.30	165	3.5	228	4.76	493	3.9
s.e	164	13.9	8.4	0.14	6.4	0.06	8.8	0.03	0.03	0.13
P-value	NS	NS	NS	NS	NS	NS	NS	NS	NS	NS

OD = once a day, TD = twice a day, ODO = once a day/outdoor.

### Experiment 2

**Figure 4:**
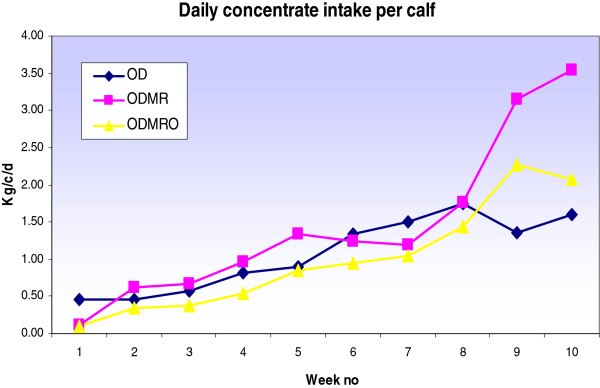
Calf intake of concentrates per day-Experiment 2.

Calf LW was higher (P < 0.05) for ODMR at day 66 and 80 compared to OD or ODMRO feeding treatments (Table 6). Calf LW at day 80 was 87, 95, and 88 kg for OD, ODMR and ODMRO, respectively. The ADG was lower (P < 0.05) for ODMRO during the period (day 25 to 38) and lower for ODMR during the period (day 39 to 52) compared to the other treatments (Table 7). However, ODMR had a higher (P < 0.001) ADG during the period (day 53–66) compared to OD or ODMRO treatments. The ADG was higher (P < 0.05) for ODMRO for the period (day 67 to 80) compared to the OD treatment. The ADG also tended to be higher (P < 0.06) for ODMR (0.79 kg) over the period (day 10 to 80) compared to OD (0.68 kg) or ODMRO (0.70 kg). The total DM intake per calf (day 10–80) was 95, 117 and 87 kg for OD, ODMR and ODMRO, respectively (Table 3). The weekly concentrate intake per calf is shown in Figure 4. Both OD and ODMRO milk feeding treatments tended to have a higher number of cases of pneumonia compared to ODMR (Table 4). The OD treatment had one respiratory related death with no deaths recorded with ODMR or ODMRO. There were no differences in the number of cases of calf diarrhoea between treatments.

**Table 6:** Effect of calf milk source type and turnout age on calf live-weight (kg) from day 10 to80 and at day410--Experiment 2

**Days**	**OD**	**ODMR**	**ODMRO**	**s**.**e**	**Sig**
10	39.1	39.7	39.3	2.0	NS
24	44.3	47.3	45.1	2.0	NS
38	53.1	55.7	51.5	2.0	NS
52	65.0	63.8	63.4	2.0	NS
66	75.5^a^	81.5^b^	72.9^a^	2.0	*
80	86.8^a^	95.1^b^	88.2^a^	2.0	*
410	293	280	276	6.6	NS

OD = once a day(whole milk), ODMR = once a day/milk replacer, ODMRO = once a day/milk replacer/outdoor.

**Table 7:** Effect of calf milk source type and turnout age on average calf daily weight gain (kg)-Experiment 2

**Days**	**OD**	**ODMR**	**ODMRO**	**s**.**e**	**Sig**
10-24	0.41	0.50	0.42	0.09	NS
25-38	0.64^a^	0.63^a^	0.48^b^	0.06	*
39-52	0.84^a^	0.56^b^	0.84^a^	0.09	*
53-66	0.82^a^	1.24^b^	0.69^a^	0.09	***
67-80	0.91^bc^	0.99^ac^	1.09^a^	0.09	*
10-80	0.68	0.79	0.70	0.03	0.06
80-410	0.62	0.56	0.57	0.02	NS

OD = once a day, ODMR = once a day/milk replacer, ODMRO = once a day/milk replacer/outdoor.

There were no differences in LW or ADG at mating (approximately 410 days) between calf rearing systems. The LW was 293, 280, and 276 kg and the ADG (day 80 to 410) was 0.62, 0.58 and 0.57 kg/d for OD, ODMR and ODMRO, respectively.

## Discussion

This study showed no difference in the parameters evaluated between cold whole milk feeding of calves either once or twice daily when calves were reared indoors. This result agrees with the previous findings of Gleeson et al. [6] who demonstrated no differences between once or twice daily feeding of cold whole milk. Furthermore, the average daily gain per calf from day 10 to 80 in this current study (0.64 kg/day) for OD calves was similar to that demonstrated by Muir et al. [15] (0.62 kg/day) with both twice-a-day and once a-day feeding.

In this present study the frequency of milk feeding did not influence the intake of concentrates when calves were reared indoors. This agrees with previous findings that concentrate intake is not affected by frequency of feeding when calves are offered concentrates ad libitum [6]. The kg of DM for milk, concentrate and hay consumed per kilogram of weight gained was 2.3 kg for OD calves and 2.2 kg for TD calves. These intakes are similar to those reported by Galton and Brakel [16] with conversion rates of 2.27 and 2.04 kg for calves fed milk replacer once and twice daily, respectively.

Allowing once daily fed calves outdoors at day 38 had an initial negative effect on calf performance, which may be due to the reduction in concentrate intake and the inclement weather conditions that prevailed at this time. Muir et al. [15] also demonstrated an improvement in performance with calves remaining indoors until 10 weeks of age on ad libitum concentrates compared to calves going to grass at 4 weeks on a restricted concentrate intake. Consumption of concentrates enables the development of the rumen necessary for the calf to digest solid feed. When a starter concentrate with adequate coarseness is fed, additional forage feeding is considered to have no added value in the early stages of a calf’s life [17].

In a previous study by Early et al. [11] calf live-weight at 63 days was similar for calves reared indoors and calves turned out to pasture at 20 days of age at which time they weighed 55 kg. In this present study calves were allowed outdoors at 38 days as they had reached an average live-weight of 52 kg . However, allowing calves outdoor at this weight and age in conjunction with weaning at 66 days resulted in lower calf LW at weaning compared to calves reared and weaned indoors.

It is suggested that extending the calf milk feeding period up to 84 days for calves fed once daily outdoors would be beneficial for future growth [1]. However, in this present study the initial average calf weight was 39 kg and extending the feeding period may be beneficial if initial calf LW were lower. While ODO calves had an average weaning LW of 67 kg this had no negative effect on subsequent calf performance. These calves however, were consuming > 1 kg of concentrate/day at approximately 6 weeks of age. This level of intake is easily achievable if access to palatable starter and water is available ad libitum [10]. Muir et al. [15] concluded that if concentrate intake was at 1 kg DM/d then calf weaning at 63 kg would have no detrimental effect on future calf performance. It could be concluded that under the management conditions employed, frequency of feeding or age at turnout had no effect on LW at mating or at post-calving or on 1^st^ lactation milk production. Average lactation milk productions (4668 kg) in this study were lower than that demonstrated by Horan et al. [18] (5533 kg) for first parity cows managed on a similar pasture based production system. However, a proportion of animals in that study were part of a high concentrate input system. A higher incidence of respiratory cases and a lower number of diarrhoea could be expected for calves reared indoors as compared to those reared outdoors [11]. In Experiment 1 no significant differences were observed for any health parameters between calves reared indoors and outdoors. However, a higher number of respiratory events and one related death were recorded for the indoor treatment OD compared to the outdoor treatment in Experiment 2.

Experiment 2: The ADG tended to be higher for ODMR over the period (day 10 to 80) compared to OD or ODMRO. The higher ADG achieved with the ODMR during this period may be due to higher concentrate consumption. Calves on the ODMR treatment had increased concentrate intake post weaning compared to ODMRO or OD. This increased consumption of concentrates explains the higher DM intake per calf compared to the OD and ODMRO feeding treatments (Table 6). However, feeding a high concentrate level indoors (2.5 kg/c/d) post weaning may have had a negative effect on calf performance when concentrate levels were reduced to 1.5 kg/c/d as calves were located outdoors on a grass diet. This may explain why the positive difference of LW (7 kg) observed for ODMR calves at day 80 was not evident at mating. The results from this study suggest that MR could be substituted for WM without any negative effects on calf performance or health. The non-pooling of WM for group feeding calves would also reduce the possible spread of disease [19]. The high performance of the MR used in this study may also be due to the high protein content which was within the range (25-27%) recommended to meet protein requirements for increasing growth rates [20].

The differences in calf LW performances between Experiment 1 and 2 for the period day 80 to 410 were related to different management factors especially during the winter period for the respective years and no comparisons could be made across experiments for this period. In both studies, heifer weights at day 410 (mating start date) were close to that as recommended to achieve maximum future animal profitability [21] and achieve a high cyclicity rate at mating [22].

## Conclusions

This study showed that calves can be successfully reared when offered either cold whole milk or milk replacer once daily from 10 days of age. Calves fed once daily indoors had similar live-weight at weaning, day 80, at breeding time and post-calving to calves fed twice daily. Milk production performance in the 1^st^ lactation was also similar for once and twice daily fed calves. Allowing calves outdoors at 38 days of age while being fed milk once daily had an initial negative effect on calf performance and this was probably due to the reduction in concentrate intake and inclement weather conditions at that time. This reduction in performance during this period did not effect subsequent live-weight performance. Calves fed milk replacer indoors had higher live-weight at both weaning and at turnout to those fed whole milk and milk replacer outdoors and this was probably due to increased concentrate intake during the post weaning period. In both studies, concentrate intake appeared to have a larger influence on calf performance to day 80 than feeding frequency or feed type. The live-weight of heifers prior to breeding appeared to be more influenced by management factors during the first winter period than during the early rearing methods. The mobile calf shelter designed for this study may be reproduced and used on commercial farms to facilitate the early turnout of calves.

## Competing interests

The authors declare that they have no competing interests.

## Authors’ contributions

DG was the lead person in the field research. BOB provided advice on study design and participated in writing of the manuscript. Both authors read and approved the final manuscript.
